# Comparative transcriptomic analysis of male and female flowers of monoecious *Quercus suber*

**DOI:** 10.3389/fpls.2014.00599

**Published:** 2014-11-06

**Authors:** Margarida Rocheta, Rómulo Sobral, Joana Magalhães, Maria I. Amorim, Teresa Ribeiro, Miguel Pinheiro, Conceição Egas, Leonor Morais-Cecílio, Maria M. R. Costa

**Affiliations:** ^1^Departamento de Recursos Naturais Ambiente e Território, Instituto Superior de Agronomia, Universidade de LisboaLisboa, Portugal; ^2^Centre for Biodiversity, Functional & Integrative Genomics, Plant Functional Biology Centre, University of MinhoBraga, Portugal; ^3^Departamento de Biologia, Faculdade de Ciências da Universidade do PortoPorto, Portugal; ^4^Biocant, Parque Tecnológico de CantanhedeCantanhede, Portugal

**Keywords:** flower development, monoecious, pyrosequencing, *Quercus suber*, RNA-seq, transcriptomics, cork oak, EST

## Abstract

Monoecious species provide a comprehensive system to study the developmental programs underlying the establishment of female and male organs in unisexual flowers. However, molecular resources for most monoecious non-model species are limited, hampering our ability to study the molecular mechanisms involved in flower development of these species. The objective of this study was to identify differentially expressed genes during the development of male and female flowers of the monoecious species *Quercus suber*, an economically important Mediterranean tree. Total RNA was extracted from different developmental stages of *Q. suber* flowers. Non-normalized cDNA libraries of male and female flowers were generated using 454 pyrosequencing technology producing a total of 962,172 high-quality reads with an average length of 264 nucleotides. The assembly of the reads resulted in 14,488 contigs for female libraries and 10,438 contigs for male libraries. Comparative analysis of the transcriptomes revealed genes differentially expressed in early and late stages of development of female and male flowers, some of which have been shown to be involved in pollen development, in ovule formation and in flower development of other species with a monoecious, dioecious, or hermaphroditic sexual system. Moreover, we found differentially expressed genes that have not yet been characterized and others that have not been previously shown to be implicated in flower development. This transcriptomic analysis constitutes a major step toward the characterization of the molecular mechanisms involved in flower development in a monoecious tree with a potential contribution toward the knowledge of conserved developmental mechanisms in other species.

## Introduction

*Quercus suber* (L.) is one of the most important forest species in Portugal, being the dominant tree of the oak woodlands (Aronson et al., 2009). Due to its ecological and socio-economic significance, the cork oak forest is a unique resource. There is a growing interest in the management of woods for the production of acorns destined either for nursery production or for animal feed stocks. Therefore, the knowledge of the molecular mechanisms that control flower induction and fertilization is crucial to fully understand the reproductive success of this species.

*Quercus suber* is a monoecious tree species with a protandrous system and a long progamic phase (period between pollination and fertilization). Male flowers are organized in catkins that emerge in reproductive buds of the previous growth season or at the base of the branches of the current season. Each individual catkin contain 15–25 staminate flowers that are radially set around the catkin's axis (Natividade, 1950). The staminate flowers present a perianth with four to six tepals with an equal or double number of anthers that do not burst simultaneously (Boavida et al., 1999). Female inflorescences arise in spikes, with three to five flowers, on the axil of the new leaves. Female flowers are included in a cupule and contain three carpels, with two ovules each (Boavida et al., 1999). Male flowering buds occur in early spring and sometimes also in autumn, whereas female flowers appear in spring and only get fully developed a few months later, if pollinated. During spike elongation, three to five styles emerge from the cupule and the stigma becomes receptive (Ducousso et al., 1993). At the time of pollination the ovary is still undifferentiated and the transmitting tissue extends only to the base of the styles. The wind driven pollen lays on the receptive stigmatic surface, germinates and the pollen tube grows throughout the transmitting tissue, until it reaches the base of the style. Usually, the pollen tube growth is arrested for 6 weeks, overlapping with ovule differentiation (Boavida et al., 1999; Kanazashi and Kanazashi, 2003). After fertilization, only one of the six ovules develops into a monospermic seed, which matures during autumn (Ducousso et al., 1993; Boavida et al., 1999).

Flower development is a complex and dynamic process that requires the tight coordination of gene expression and environmental cues (Fornara et al., 2010). During the past several years, a significant progress has been made in elucidating the genetic networks involved in flower organ specification in hermaphroditic model (reviewed in Wellmer et al., 2014) and non-model species (Wu et al., 2010; Yoo et al., 2010; Zahn et al., 2010; Logacheva et al., 2011; Varkonyi-Gasic et al., 2011; Zhang et al., 2012). Unisexual flower specification requires developmentally regulated processes that initiate male and female organ primordia in separate parts of the plant (Dellaporta and Calderon-Urrea, 1993). Studies focusing on mutant isolation revealed that several genes affect the key steps of sex determination in a variety of species. For example, in maize, unisexuality is controlled by *TASSELSEED2* that is expressed in the male structure (tassel) and is involved in pistil primordia abortion (DeLong et al., 1993). Also, in melon, a single nucleotide change in the *1-AMINOCYCLOPROPANE-1-CARBOXYLIC ACID SYNTHASE* gene is responsible for the specific inhibition of the male reproductive organs (Boualem et al., 2008). With the advent of next generation sequencing (NGS) technology, the previous limitation of mutant isolation in important model and non-model species was surpassed (Rowan et al., 2011). In another Cucurbitaceae, prior knowledge established a link between the de-regulation of the homeotic ABC model genes and sex determinacy (Kater et al., 2001). Using NGS technology, Guo et al. (2010) further helped to understand the molecular mechanisms underlying sex determinacy in cucumber by comparing the transcriptomes of the two types of flowers (gynoecious and hermaphroditic). In *Quercus* spp., many studies have been conducted focusing on the morphology of reproductive organs (Kaul, 1985), life cycle (Ducousso et al., 1993; Elena-Rossello et al., 1993), flowering process (Varela and Valdiviesso, 1996), and embryogenesis (Stairs, 1964). However, molecular information regarding these mechanisms is still scarce. Ueno et al. (2010) described the first large-scale study of bud transcriptomes of the two main European white oak species (*Q. petraea* and *Q. robur*). Ueno et al. (2013) used the same pyrosequencing technology to characterize the bud transcriptomes of endo- and ecodormant sessile oak (*Q. petraea*). Recently, the transcriptome of *Q. suber* has been reported using 21 normalized cDNA libraries derived from multiple *Q. suber* tissues and organs, developmental stages and physiological conditions (Pereira-Leal et al., 2014). This work included two normalized libraries of *Q. suber* (male and female) flowers that could serve as a tool to mine genes in each flower type. However, data concerning differentially expressed genes during different developmental stages of each type of flower were still missing.

In the present work, with the aim of capturing the diversity of transcripts differentially expressed in male and female *Q. suber* flowers, inflorescences in different developmental stages were separately collected and non-normalized cDNA libraries were generated and sequenced using the 454 GS-FLX Titanium technology. This study provides a unique set of databases, invaluable for gene discovery, which might reveal the regulatory networks of sex-specific flower development of a non-model monoecious tree species.

## Materials and methods

### Plant material

**Figure 1:**
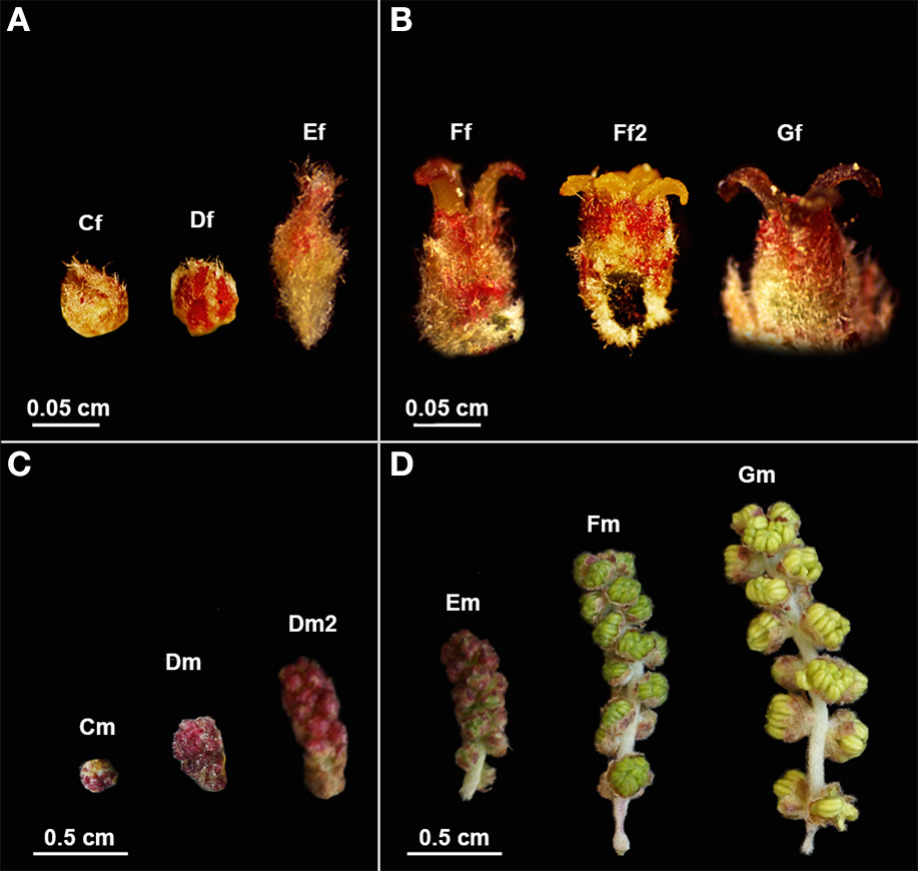
***Quercus suber* female and male flowers in different developmental stages used in RNA-seq. (A)** Early and **(B)** late stages of female flower development used in pools 1F and 2F, respectively. **(C)** Early and **(D)** late stages of male flower development used in pools 1M and 2M, respectively. (Cf) female bud enclosed by protective scales; (Df) female reddish bud with open scales; (Ef) elongation of the spike axe and the emergency of the first pair of flowers; (Ff) female flower showing distinct, erect, yellow stigmas with curved pinkish/brownish tips; (Ff2) flower with shining yellow and viscous pattern stigmas in clear divergent position; (Gf) female flower with closed stigmas that lost the receptivity, exhibiting a dark brown color. (Cm) catkin with red round shape due to the tight clustering of the flowers; (Dm) elongated cluster of male flowers; (Dm2) pendent catkin with some individualized flowers; (Em) male flowers with the anthers individualized; (Fm) flowers with individualized green/yellow anthers where pollen shedding begins; (Gm) catkin with male flowers in full anthesis.

Six developmental stages of male and female cork oak flowers were collected from different trees in three different locations in Portugal (Lisbon, Porto, and Braga). The classification of the different phenological phases was based on visual observation, according to Varela and Valdiviesso (1996) (Figure 1). Samples were harvested between the end of March and the beginning of June and were frozen in liquid nitrogen immediately after collection.

### RNA extraction and cDNA preparation

RNA was extracted from each sample using the RNAqueous® Kit (Ambion), following the manufacturer's instructions. The same amount of RNA was combined to create four specific RNA pools, two for female flowers (1F and 2F) and two for male flowers (1M and 2M), covering either early (1F or 1M) or late (2F or 2M) developmental stages. Pool 1F (Figure 1A) contained RNA from female buds enclosed by protective scales (Cf), female reddish buds with open scales (Df) and buds showing the elongation of the spike axe and the emergency of the first pair of flowers (Ef). The 2F pool (Figure 1B) included RNA from female flowers showing distinct, erect, yellow stigmas with curved pinkish/brownish tips (Ff), flowers with shining yellow and viscous pattern stigmas in clear divergent position (Ff2) and flowers with closed stigmas that lost the receptivity, exhibiting a dark brown color (Gf). The 1M pool (Figure 1C) comprised RNA from catkins with red round shape (Cm), elongated clustered male flowers (Dm), and pendent catkins with some individualized flowers (Dm2). The 2M pool (Figure 1D) included male flowers in which the anthers were becoming individualized (Em), flowers with individualized green/yellow anthers, prior to pollen shedding (Fm) and catkins with male flowers in full anthesis (pollen shedding occurring in half of the flowers with some anthers eventually empty) (Gm). RNA integrity was verified on an Agilent 2100 Bioanalyzer with the RNA 6000 Pico kit (Agilent Technologies) and the quantity assessed by fluorometry with the Quant-iT RiboGreen RNA kit (Invitrogen). A fraction of 2.0 μ g of each pool of total RNA was used as starting material for cDNA synthesis using the MINT cDNA synthesis kit (Evrogen), where a strategy based on SMART double-stranded cDNA synthesis (Zhu et al., 2001) was applied. During the amplification of the poly RNA molecules, a known adapter sequence was introduced to both ends of the first strand of cDNA. The synthesis was also performed using a modified oligo-dT, containing a restriction site for *Bsg*I that is needed to eliminate the tails, to minimize the interference of homopolymers during the 454-sequencing run. cDNA was quantified by fluorescence and sequenced in a full plate of 454 GS FLX Titanium system, according to the standard manufacturer's instructions (Roche-454 Life Sciences) at Biocant (Cantanhede, Portugal). Sequence reads were deposited in the NCBI Sequence Read Archive (SRA) under the accession number SRP044882.

### Sequence processing assembly and annotation

Prior to the assembly of sequences, the raw reads were processed in order to remove sequences with less than 100 nucleotides and low-quality regions. The ribosomal, mitochondrial and chloroplast reads were also identified and removed from the data set. The reads were then assembled into contigs using 454 Newbler 2.6 (Roche) with the default parameters (40 bp overlap and 90% identity).

The translation frame of contigs was assessed through BLASTx searches against Swissprot (*e*-value = 1e–6), and the corresponding amino acid sequences translated using an in-house script. Next, the contigs without translation were submitted to FrameDP software (Gouzy et al., 2009) and the remaining contigs were analyzed with ESTScan (Lottaz et al., 2003). Transcripts resulting from these two last sequence identification steps (FrameDP and ESTScan) were searched using BLASTp against the non-redundant NBCI (National Center for Biotechnology Information) database in order to translate the putative proteins.

The deduced aminoacid sequences were annotated using InterProScan (Hunter et al., 2009) and each was given the Gene ontology terms (GOs) (Ashburner et al., 2000).

To identify the differential gene expression between samples, the contigs were clustered using the CD-Hit 454 (Niu et al., 2010) application (90% similarity) in order to eliminate redundant sequences and generate reference contigs. After this step, the contigs that codify non-redundant proteins were used as reference to map the reads. The mapping process was made using 454 Newbler Mapping 2.6 (Roche). The mapping results were quantified to obtain the number of reads from different samples and a contingency table with contig names was created using the number of reads per reference contig per sample. The contingency table was normalized at a 95 percentile using the MyRNA (Langmead et al., 2010) statistical analysis package and the differential gene expression was evaluated using a linear regression model based on a Gaussian distribution and taking into account only contigs with a minimum of eight mapped reads and FDR < 0.05. Differentially expressed were clustered using the Self-organizing Trees algorithm (SOTA), euclidean distance (Dopazo and Carazo, 1997; Herrero et al., 2001) and the default settings of the MeV: MultiExperiment Viewer program (http://www.tm4.org/mev.html).

### qRT-PCR analysis

cDNA was synthesized from the same RNA samples used for the 454 sequencing, according to the manufacturer's instructions. cDNA was amplified using SsoFast™ EvaGreen® Supermix (Bio-Rad), 250 nM of each gene-specific primer (listed in Supplementary Table S1) and 1 μ L of cDNA (1:100 dilution). Quantitative real-time PCR (qRT-PCR) reactions were performed in triplicates on the CFX96 Touch™ Real-Time PCR Detection System (Bio-Rad). After an initial period of 3 min at 95°C, each of the 40 PCR cycles consisted of a denaturation step of 10 s at 95°C and an annealing/extension step of 10 s at the gene specific primer temperature. With each PCR reaction, a melting curve was obtained to check for amplification specificity and reaction contaminations, by heating the amplification products from 60°C to 95°C in 5 s intervals. Primer efficiency was analyzed with CFX Manager™ Software v3.1 (Bio-Rad), using the Livak calculation method for normalized expression (Livak and Schmittgen, 2001). Gene expression analysis was established based on three technical and biological replicates, and normalized with the reference gene *QsPP2AA3* (Marum et al., 2012).

## Results and discussion

Due to the large number of reads attainable, the 454 DNA sequencing technology has great potential for discovering transcripts in non-model organisms. A prior study by Pereira-Leal et al. (2014) provided the first step toward the assembly of the monoecious tree *Q. suber* transcriptome using normalized libraries. In order to capture the diversity of transcripts differentially expressed during the development of female (F) and male (M) flowers, different developmental stages of flowers were collected covering either early (1F and 1M) or late (2F and 2M) developmental stages (Figure 1).

### Sequencing and assembly of *Q. suber* flower transcriptome

Pyrosequencing resulted in 332,607 (1F), 312,282 (2F), 255,962 (1M), 270,871 (2M) raw reads for each library. After trimming, a total of 280,092 (1F), 252,024 (2F), 205,781 (1M), 224,275 (2M) high-quality reads were available with an average length of 264, 253, 269, and 270 bp, respectively (Table 1). Reads were assembled into 7565, 6923, 5267, and 5171 contigs in 1F, 2F, 1M, and 2M, respectively, with an average contig length of 714 to 812 bp (Table 1).

**Table 1:** **Sequencing and annotation statistics of *Quercus suber* flower libraries**.

	**1F**	**2F**	**1M**	**2M**	**1F_1M_2F_2M**
Number of raw reads	332,607	312,282	255,962	270,871	
Number of reads after trimming	280,092	252,024	205,781	224,275	837,163
Average read length after trimming	264	253	269	270	263
Number of contigs	7565	6923	5267	5171	16,832
Average contig length	773	714	779	812	914
Range of contig length	[60.. 3489]	[52.. 3394]	[36.. 3384]	[15.. 3392]	[16.. 3848]
Number of translated contigs	7289	6600	5090	4981	16,152
Peptide sequences with BLASTx matches	5723	5154	4083	3997	11,956
Peptide sequences translated by FrameDP	2211	1907	1386	1398	6621
Peptide sequences translated by ESTScan	108	117	116	114	297
Total of amino acid sequences	8042	7178	5585	5509	18,874
Peptide sequences with BLASTP matches	1294	1086	809	825	3372
Amino acid sequence assigned InterPro terms	5940	5312	4251	4217	12,698
Amino acid sequence assigned to GO terms	4536	4056	3272	3269	9459

*Individual libraries were generated from four specific RNA pools, two for female flowers (1F and 2F) and two for male flowers (1M and 2M), covering either early (1F and 1M) or late (2F and 2M) stages of flower development. The four individually EST projects were assembled into the 1F_1M_2F_2M library. The libraries were assembled using 454 Newbler 2.6 (Roche) and annotated in a three-step process using BLASTx search, FrameDP and ESTScan using default parameters*.

High-quality reads from the four individual EST libraries were assembled together into a single library (1F_2F_1M_2M), generating 837,163 high-quality reads with an average length of 263 bp that were assembled into 16,832 contigs with an average contig length of 914 bp (Table 1).

**Figure 2:**
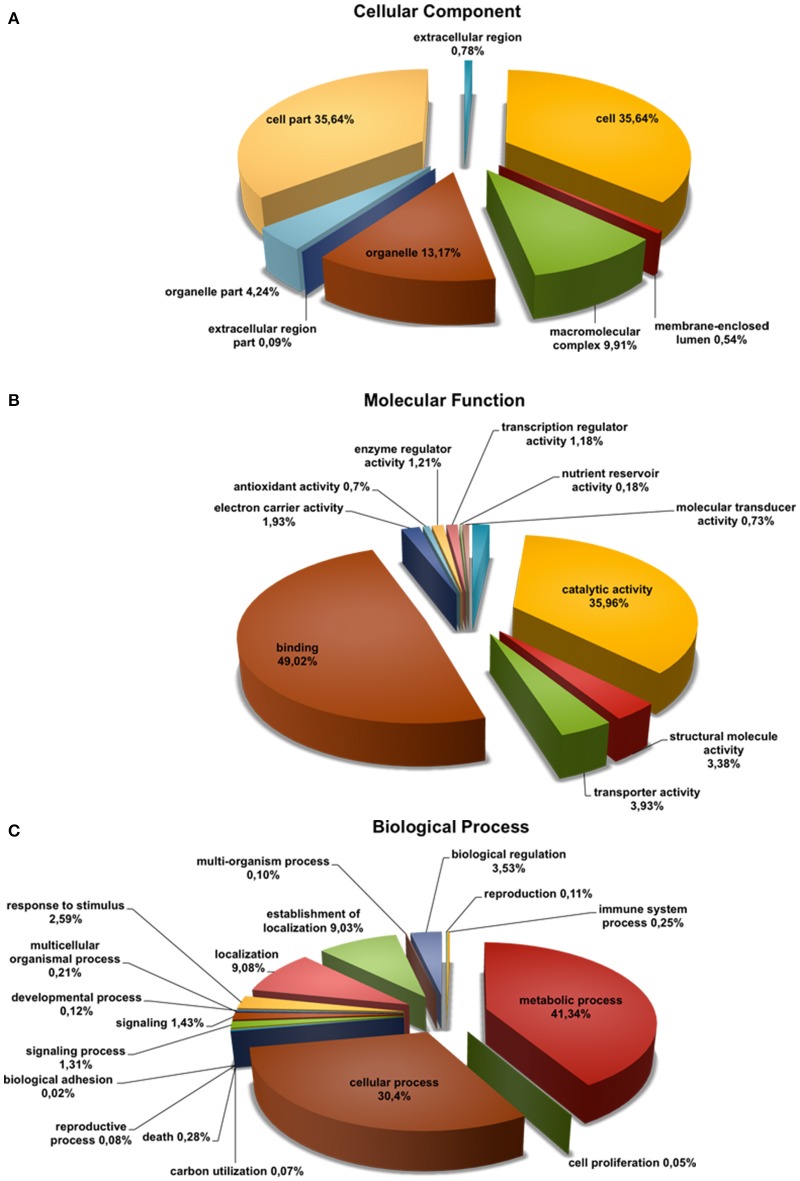
**Functional classification of *Quercus suber* unigenes**. Four EST projects were generated from four-specific RNA pools, two for female flowers (1F and 2F) and two for male flowers (1M and 2M), covering either early (1F and 1M) or the late (2F and 2M) developmental stages. The four individually EST projects were assembled into the 1F_1M_2F_2M library and the deduced aminoacid sequences of this library were annotated using InterProScan. The Gene Ontology terms (GOs) for each translated amino acid sequence were used to classify the transcript products within the category of **(A)** cellular component, **(B)** molecular function, and **(C)** biological process sub-ontologies.

To annotate the *Q. suber* flower transcriptome, a three-step process (BLASTx search, FrameDP and ESTScan) was performed resulting in 16,152 (95.96%) translated contigs for the 1F_2F_1M_2M library (Table 1). GO terms were then assigned, indicating a total of 9459 aminoacid sequences (50%) with at least one GO term (Table 1). Based on the GO annotations, cell, metabolic process and binding were the most abundant GO slims within the cellular component, molecular function, and biological process categories, respectively (Figure 2). Metabolic process (41.34%) and cellular process (30.40%) were the most highly represented groups within the biological process category, indicating that the floral tissues were undergoing extensive physiological activity in accordance with what was observed in *Arabidopsis thaliana* reproductive tissues (Hennig et al., 2004).

### Validation of the *Q. suber* flower transcriptomes

The *in silico* analysis of the transcriptomes allowed the identification of differences between the distinct developmental stages of male and female flowers. In order to validate the differences observed between male and female flower libraries, several contigs were identified by homology with functional important genes known to be involved in carpel or stamen development in model organisms. Homologs for *ABORTED MICROSPORES* (*AMS*, Xu et al., 2010), *LESS ADHERENT POLLEN3* (*LAP3*, Dobritsa et al., 2009), *LESS ADHESIVE POLLEN5* (*LAP5*, Dobritsa et al., 2010), and *LESS ADHESIVE POLLEN6* (*LAP6*, Dobritsa et al., 2010) were chosen as the male candidate genes due to their involvement in pollen development. Homologs for the female candidate genes, *At4g27290* (Pagnussat et al., 2005), *CYTOCHROME P450 78A9* (*CYP78A9*, Ito and Meyerowitz, 2000), *POLYGALACTURONASE-1* (*PG1*, Tacken et al., 2010), and *STIGMA SPECIFIC1* (*STIG1*, Verhoeven et al., 2005) were selected based on their relevance in pollen recognition, stigma and transmitting tract development.

**Figure 3:**
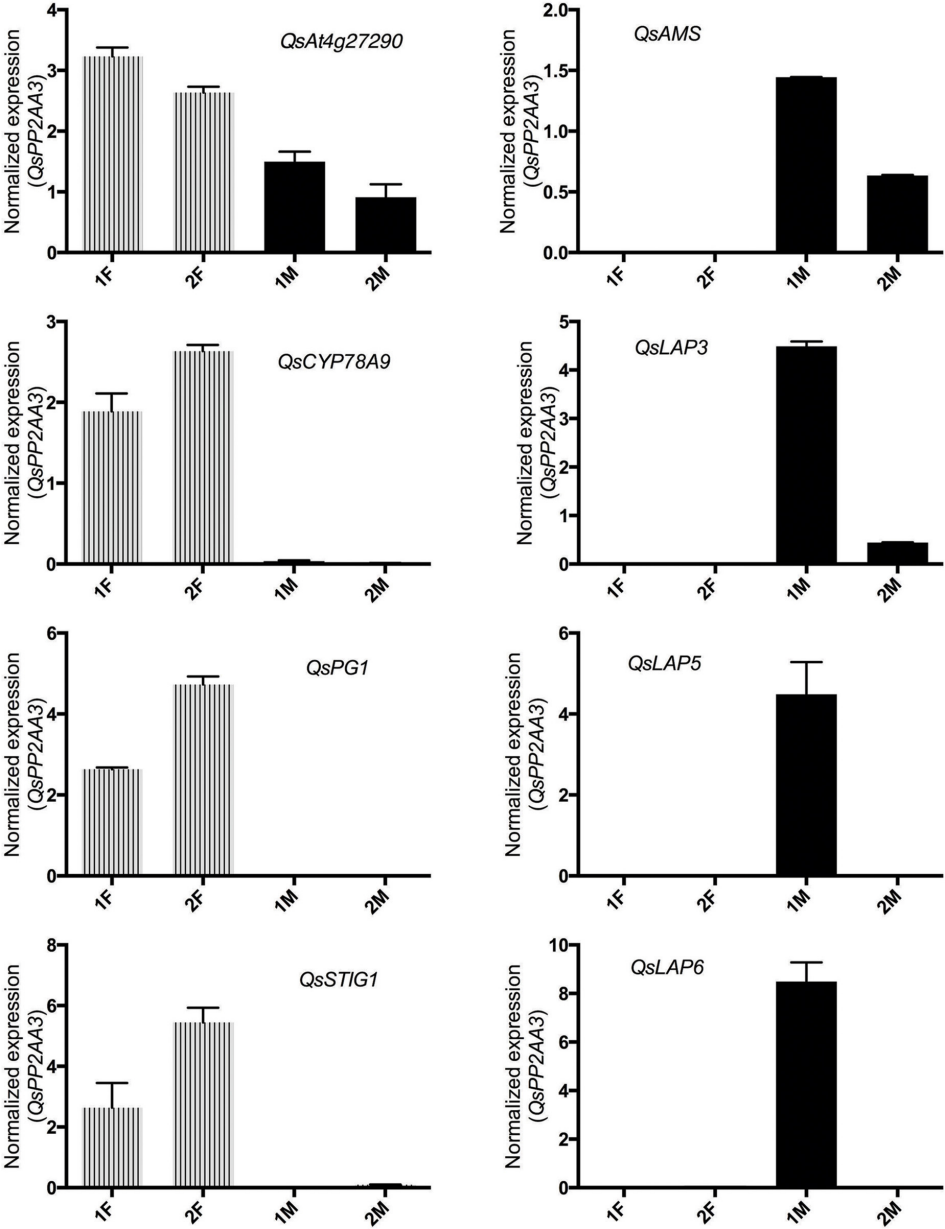
**Relative expression of differentially expressed male and female genes chosen to validate RNAseq results**. *QsAMS, QsLAP3, QsLAP5, QsLAP6* were selected as male candidate genes, whereas *QsAt4g27290, QsCYP78A9, QsPG1*, and *QsSTIG1* as female candidate genes. Transcript abundance was determined using qPCR, and normalized to *QsPP2AA3* using cDNA synthesized from distinct pools of RNA covering either early (1F and 1M) or late (2F and 2M) stages of male or female flower development. Reactions were performed in three biological and technical replicates. Error bars indicate standard deviation (SD).

As expected, the *Q. suber* homologous genes presented differential expression ratios between male and female libraries (Table 2), and thus were considerate good candidates for qRT-PCR analysis. The qRT-PCR results confirmed that genes involved in pollen exine formation (*LAP3, LAP5*, and *LAP6*) and in the tapetum cell development (*AMS*) were more expressed in the early stages of male flower development (Figure 3), whereas genes involved in stigma-specific recognition (*STIG1*), in the recognition of pollen (*At4g27290*) and in fruit growth and development (*CYP78A9, PG1*) were more expressed in the female flowers. These results were in close agreement with the RNAseq data (Table 2) suggesting the reliability of the transcriptomic profiling data.

**Table 2:** **Candidate genes that were selected to validate the transcriptional levels determined by RNAseq results**.

**Candidate genes**	**Gene accession**	**1F**	**2F**	**1M**	**2M**
• *At4g27290*	QSP078589.0	29	14	0	1
• *CYTOCHROME P450 78A9*	QSP091316.0	70	70	0	0
• *POLYGALACTURONASE1*	QS094531.0	19	337	0	0
• *STIGMA SPECIFIC1*	QS121989.0	5	34	0	0
* *ABORTED MICROSPORES*	QS049646.0	0	0	38	4
* *LESS ADHERENT POLLEN3*	QS049611.0	1	2	170	46
* *LESS ADHESIVE POLLEN5*	QS003695.0	0	0	566	6
* *LESS ADHERENT POLLEN6*	QS039918.0	0	0	924	49
*PROTEIN PHOSPHATASE 2A SUBUNIT A3*	QS092015.0	55	71	57	65

*The number of ESTs represents their distribution in the female and male Quercus suber combined flower library, covering either early (1F and 1M) or late (2F and 2M) stages of flower development. The selected genes were identified by homology with functional important genes known to be involved in carpel or stamen development*.

• *Female candidate genes*.

* *Male candidate genes*.

*PROTEIN PHOSPHATASE 2A SUBUNIT A3 was chosen as the reference gene. Gene accessions according to CorkOak database (www.corkoakdb.org)*.

### Differential gene expression between *Q. suber* flower-type libraries

**Figure 4:**
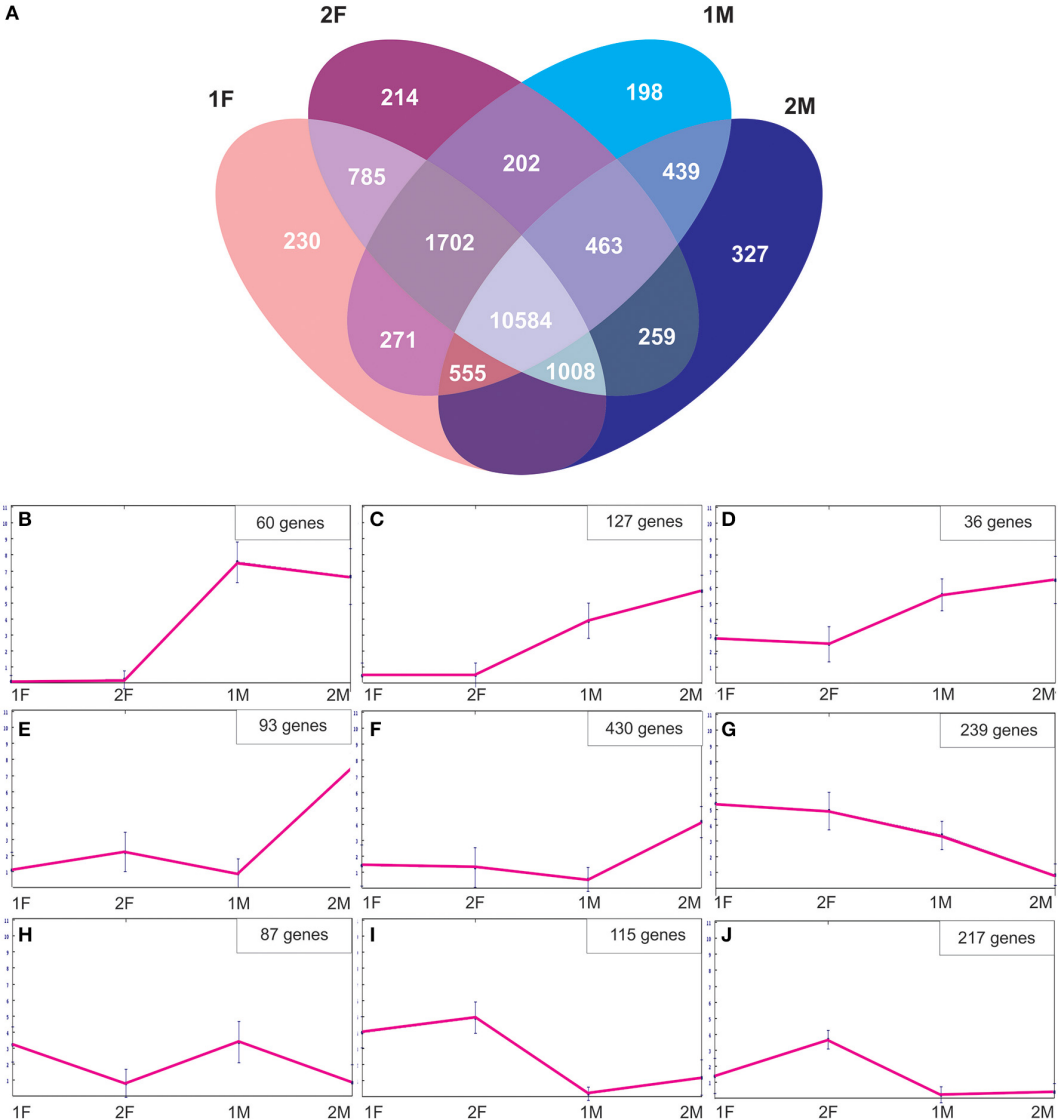
**Description of the *Quercus suber* unique and differentially expressed genes. (A)** Venn diagram indicating the number of exclusive and shared transcripts of early and late developmental stages of *Quercus suber* flower. Four EST projects were generated from four-specific RNA pools, two for female flowers (1F and 2F) and two for male flowers (1M and 2M), covering either early (1F and 1M) or the late (2F and 2M) developmental stages. The four individually EST projects were assembled into the 1F_1M_2F_2M library and the exclusive transcripts were identified using the Venny application (Oliveros, 2007). **(B–J)** Differentially expressed genes were clustered using the Self-organizing Trees algorithm (SOTA), euclidean distance (Dopazo and Carazo, 1997; Herrero et al., 2001) and the default settings of the MeV, MultiExperiment Viewer program (http://www.tm4.org/mev.html).

In order to identify exclusive transcripts of early and late developmental stages of female and male flower development, the assembly of the four non-normalized libraries was analyzed. The analysis showed that there were 230 unique contigs for the early (1F) and 214 contigs unique for the late (2F) stages of female flower development (Figure 4A). The 1F unique contigs might correspond to genes controlling early flower development, whereas the 2F unique contigs might be associated with stigma maturation, ovule development and fertilization. Accordingly, there were 198 contigs unique in the early stages of male flower development (1M), most probably involved in early stages of anther development and 327 contigs specific for the late stages (2M) that could be indicative of genes controlling pollen development and maturation (Figure 4A). A normalization cut-off of eight reads at the 95th percentile was applied, resulting in 3760 differentially expressed genes (19.9%) for the 1F_2F_1M_2M transcriptome. Differentially expressed genes were then clustered into different groups according to their expression profile similarity (Figure 4). Groups of genes that were either unique (Figure 4B) or significantly more expressed in the male samples (Figures 4C,D) were identified. At least 430 differentially expressed genes were predominantly expressed in the last stages of male flower development (Figures 4E,F), whereas 239 genes were absent from this stage and present in all the other libraries (Figure 4G). We also found genes that were more expressed in the early stages of both male and female flower development (Figure 4H). A group of genes (115) was more expressed in both female libraries (Figure 4I), whereas 217 genes appear to be preferentially expressed in late stages of female flower development (Figure 4J).

Out of the 3760 differentially expressed genes, a GO term was assigned to 1797 female and to 745 male transcripts. No significant differences were found between male and female GO categories apart from the molecular and cellular functions. In the former, 16% of the female GO terms were assigned to protein binding in contrast with the male GO terms (7%), whereas in the latter, 8% of the female GO terms were assigned to the nucleus and just 1% of the male were appointed to the nucleus (Figure S1).

### Potential allergen genes present in *Q. suber* libraries

During spring, Fagales tree species produce and release large amounts of pollen. In Southern Europe, pollen from these plants and other anemophilous trees, like *Platanus acerifolia* and *Olea europaea*, has been proved to elicit allergic diseases, such as pollinosis rhinitis/rhino conjunctivitis (D'Amato et al., 2007; Esteve et al., 2012). The official site for the systematic allergen nomenclature (http://www.allergen.org), that was approved by the World Health Organization and International Union of Immunological Societies (WHO/IUIS) Allergen Nomenclature Sub-committee, lists 263 allergenic proteins to the taxonomic group Plantae Magnoliopsida. Among these 263 allergens, 34 were associated with the Fagales order and only one (Quea1) was related to the genus *Quercus*.

In order to identify transcripts encoding potential allergens in cork oak, blast searches were carried on the 1F_1M_2F_2M transcriptome against the proteins reported as allergenic and included in the WHO–IUIS list. This analysis revealed several potential orthologs for genes coding for potential allergens in *Q. alba, Betula pendula, Corylus avellana, O. europaea, Hevea brasiliensis*, and *P. acerifolia* (Table 3). Of major interest was the identification of a potential ortholog of Quea1, which is the major allergen of *Quercus alba* (Wallner et al., 2009). As expected, almost all the potential orthologs of allergen genes were highly expressed in the male libraries (Table 3).

**Table 3:** ***Quercus suber* potential allergens in flower transcriptome**.

**Gene designation**	**Gene accession**	**ESTs 1F + 2F**	**ESTs 1M + 2M**	**Allergen**	**Closest species homolog**	**InterProScan description**
*QsQUEA1*	QS091157.0	11	367	Quea1	*Quercus alba*	Pollen allergen Bet v 1
*QsBETV2*	QS034447.0	2	22	Betv2	*Betula pendula*	Profilin
*QsBETV3*	QSP068142.0	4	26	Betv3	*Betula pendula*	Calcium-binding allergen Bet v 3
*QsBETV6*	QSP015348.0	40	100	Betv6	*Betula pendula*	Isoflavone reductase homolog
*QsCyP*	QS017405.0	274	183	Betv7	*Betula pendula*	Cyclophilin
*QsBETV4*	QS115544.0	0	65	Betv4	*Betula pendula*	Polcalcin Bet v 4
*QsBIP*	QS126407.0	380	703	Cora10	*Corylus avellana*	Luminal binding protein
*QsOLE1*	QS069793.0	1	89	Olee1	*Olea europae*	Ole e1-like protein
*QsOLE9*	QS095617.0	9	248	Olee9	*Olea europae*	β- 1.3 Glucanase
*QsPME*	QS101292.0	1	72	Olee11	*Olea europea*	Pectin methyl esterase
*QsSOD_CU_ZN*	QS057690.0	115	96	Olee5	*Olea europea*	Superoxide dismutase. copper/zinc binding
*QsHEV1*	QS150670.0	12	11	Hevb6	*Hevea brasiliensis*	Pro-hevein
*QsPLAA2*	QS060812.0	5	311	Plaa2	*Platanus acerifolia*	Exopolygalacturonase
*QsnsLTP*	QS106891.0	1097	3077	Plaa3	*Platanus acerifolia*	Non-specific lipid-transfer protein

*The number of ESTs represents their distribution in the female (1F+2F) and male (1M+2M) Q. suber combined flower library. Selected genes were identified by homology with functional important allergen genes included in the WHO-IUIS allergen list. The deduced aminoacid sequences of these genes were annotated using InterProScan. Gene accessions according to CorkOak database (www.corkoakdb.org)*.

### *Q. suber* most differentially expressed genes between female and male tissues

The ten genes most differentially expressed in both male and female tissues were identified by establishing a ratio between male and female EST counts (Table 4). Concerning the differentially expressed genes more represented in female flowers, we identified a homolog for *POLYGALACTURONASE-1* that is comparatively 356 times more expressed in female tissues. Interestingly, several studies report the involvement of polygalacturonases associated genes to both carpel (Ogawa et al., 2009) and pollen development (Allen and Lonsdale, 1993; Tebbutt et al., 1994; Rhee et al., 2003). *QsENDO-BETA-1,3-1,4 GLUCANASE*, a member of the glycoside hydrolase family, is 199 times more expressed in female samples. In *Populus trichocarpa*, a member of this family, *PtrCel9A6*, is tightly involved in sexual determinism (Yu et al., 2013). Overexpression of *PtrCel9A6* in *A. thaliana* resulted in male sterility due to defects in anther dehiscence (Yu et al., 2013). It is possible that the *QsENDO-BETA-1,3-1,4 GLUCANASE* might have a similar function by inhibiting the development of male structures in female flowers.

**Table 4:**
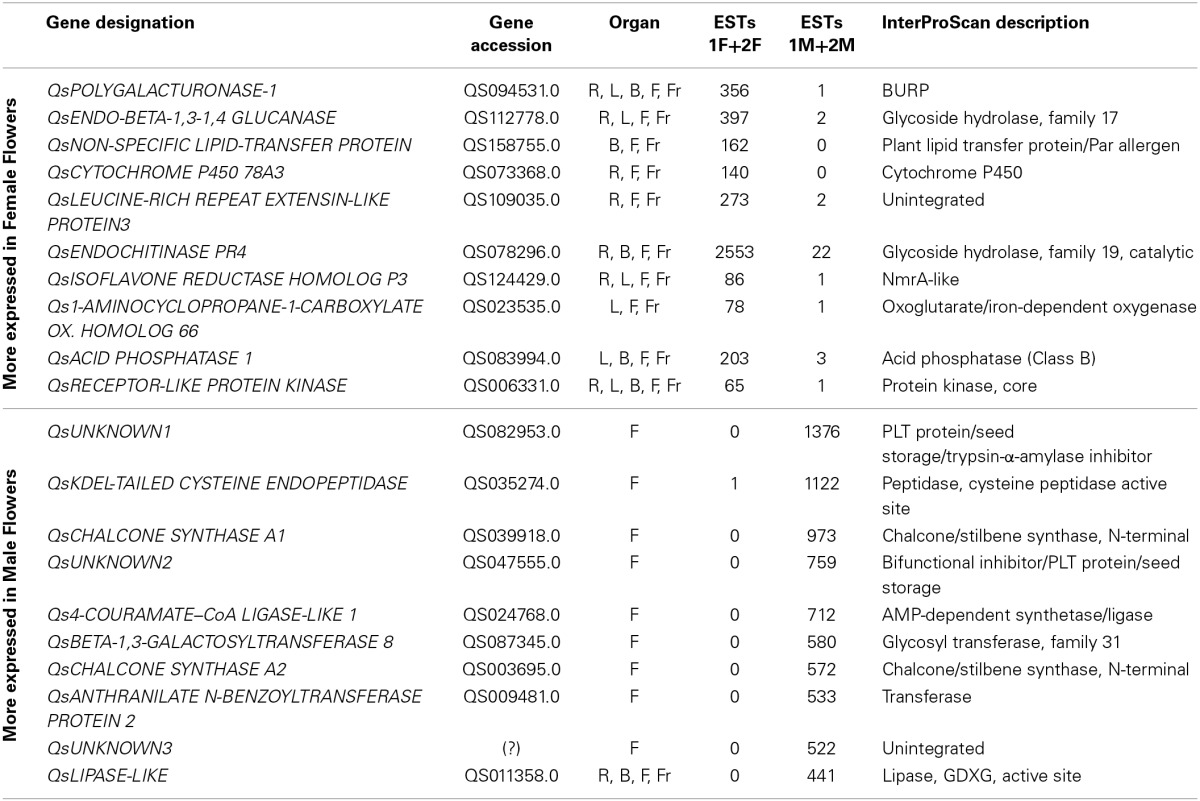
***Quercus suber* most differentially expressed genes in the flower transcriptome**.

*The 10 genes most differentially expressed in male or female tissues were identified by establishing a ratio between male and female EST count in female (1F+2F) and male (1M+2M) Q. suber combined flower library. The deduced aminoacid sequences of these genes were annotated using InterProScan. Gene accessions according to the CorkOak database [www.corkoakdb.org; (?) not present]. Transcript distribution of the most differentially expressed genes in the Q. suber organs was obtained by blasting the genes sequences against the organ libraries made available by Pereira-Leal et al. (2014). R, root; L, leaf; B, bud; F, flower; Fr, Fruit*.

A *CYTOCHOME P450* transcript (*QsCYTOCHROME P450 78A3*) was also highly represented in the female samples with a possible role in carpel gametophyte and sporophyte development as it was shown for homologous genes in other species (Ito and Meyerowitz, 2000; Chakrabarti et al., 2013). Ito and Meyerowitz (2000) identified *AtCYP450 78A9*, a gene that when overexpressed in *A. thaliana* results in altered fruit and seed. Another member of this family is *SlKLUH*, which controls not only plant architecture but also fruit mass and ripening in tomato (Chakrabarti et al., 2013). We also identified a homolog for *1-AMINOCYCLOPROPANE-1-CARBOXYLATE OXIDASE 66*, that belongs to a family of genes that has been associated to ethylene biosynthesis (Barry and Giovannoni, 2007). Several *RECEPTOR-LIKE PROTEIN KINASES* (*RLPK*) have been linked to key aspects of plant development: the brassinosteroids signaling pathway (Schumacher and Chory, 2000), meristem maintenance (Clark, 2001), or pollen-pistil interaction (McCubbin and Kao, 2000). Thus, a 65 times more expressed *QsRLPK* transcript in female flowers might have an important role in carpel development.

Within the group of genes with the highest differential expression in male flowers, there were three unknown genes without a significant BLAST hit. These three genes may be specific to *Q. suber* and pivotal to anther differentiation and development in this species. Two highly represented *QsCHALCONE SYNTHASE A* genes were identified as male unique transcripts. A suppressor mutant of the *CHALCONE SYNTHASE A* homolog gene in *Petunia* generates viable pollen, however, pollen germination and tube growth are severely affected (Taylor and Jorgensen, 1992), which indicates that this gene is essential for proper anther development. *CHALCONE SYNTHASE A* associated genes are also known to be involved in the metabolic pathway that leads to the production of flavonoids and anthocyanin pigments in several species (Winkel-Shirley, 2002). Thus, it is possible that the coloration on the anthers might be due to the action of the highly expressed *QsCHALCONE SYNTHASE A* genes. A *Qs4-COURAMATE–CoA LIGASE-LIKE 1* gene that is related to the *ACOS* genes was also identified. These genes have been associated with proper pollen development (De Azevedo Souza et al., 2009). The *DEFECTIVE IN ANTHER DEHISCENCE1* (*DAD1*) is a lipase-like gene involved in pollen development (Ishiguro et al., 2001). In *A. thaliana dad1* shows defects in anther dehiscence, pollen maturation, and flower opening (Ishiguro et al., 2001). *QsLIPASE-LIKE* might also have a similar function.

To investigate whether these genes were flower specific, available root, leaf, bud and fruit libraries (Pereira-Leal et al., 2014) were used. Interestingly, all the male-associated genes are exclusive to the male flower except for *QsLIPASE-like*, which is expressed in almost all the other tissues with the exception of the female flower and leaves (Table 4). The majority of the female-associated genes analyzed are not flower specific. Interestingly, there is one gene that is present only in the buds, flowers and fruits (*QsNON-SPECIFIC LIPID-TRANSFER PROTEIN*), suggesting a putative role in the female reproductive determinism. At least two genes are expressed in all the organs (*QsPOLYGALACTURONASE-1* and *QsRECEPTOR-LIKE PROTEIN KINASE*) except for the male flowers. Considering that these genes are expressed in all *Q. suber* tissues analyzed and absent from the male flowers might be indicative that these flowers go through a very distinctive developmental programme, or that the mentioned genes expression could be detrimental to proper male flower development. It will be very interesting to perform functional studies to analyse the involvement of the aforementioned genes in plant reproduction or flower development in *Q. suber* and other flowering species.

### Transcription factors differentially expressed in female and male flowers

Differential expression of transcription factors (TF) has a pivotal role in the control of mechanisms that direct organ development (Latchman, 1997). Based on the analysis of the different TF groups, a group of biologically interesting genes that are sex-specific or differentially expressed in each library was identified (Table 5).

**Table 5:** **Transcription factors differentially expressed in male and female flowers of *Quercus suber***.

**Gene designation**	**Gene accession**	**Organ**	**ESTs 1F + 2F**	**ESTs 1M + 2M**	**InterProScan description**
*QsCONSTANS LIKE 9*	QS124225.0	F,L	34	7	Zinc finger, B-box
*QsCONSTANS LIKE 4*	QS050989.0	R, L, B, F, Fr	270	68	Zinc finger, B-box
*QsCONSTANS LIKE 5*	QS116713.0	R, L, B, F, Fr	82	35	Zinc finger, B-box
*QsTRANSPARENT TESTA 1*	QS064811.0	R, L, B, F, Fr	38	4	Zinc finger, C2H2-type
*QsABORTED MICROSPORES*	QS049646.0	F	0	42	bHLH dimerization region
*QsBRASSINOSTEROID ENHANCED EXPRESSION1*	QS048556.0	F	39	0	bHLH dimerization region
*QsINDUCER OF CBF EXPRESSION1*	QS129356.0	R, L, B, F, Fr	38	4	bHLH dimerization region
*QsGLABRA3*	QS150704.0	B, F Fr	31	5	bHLH dimerization region
*QsMYC2*	QS073124.0	R, L, B, F, Fr	114	26	bHLH dimerization region
*QsIAA-LEUCINE RESISTANT3*	QS154009.0	R, L, B, F, Fr	83	39	bHLH dimerization region
*QsPERIANTHIA*	QS095157.0	F	39	15	Basic-leucine zipper (bZIP)
*QsCUC1*	QS061789.0	R, F, Fr	33	6	No apical meristem (NAM) protein
*QsCUC2*	QS009784.0	R, F, Fr	33	7	No apical meristem (NAM) protein
*QsIAA27*	QS075617.0	R, L, B, F, Fr	110	17	AUX/IAA protein
*QsIAA9*	QS117343.0	R, L, B, F, Fr	160	97	AUX/IAA protein
*QsPIN1*	QS117199.0	R, B, F, Fr	20	1	Auxin efflux carrier
*QsEIN3*	QS119163.0	R, F	18	1	Ethylene insensitive 3
*QsMYB33*	QS020061.0	F	0	13	Homeodomain-like
*QsSUPRESSOR OF CONSTANS1*	QS149164.0	R, L, B, F Fr	25	2	Transcription factor, MADS-box
*QsAP1*	QS003005.0	F Fr	68	8	Transcription factor, MADS-box
*QsFRUITFULL*	QS029922.0	F Fr	53	7	Transcription factor, MADS-box
*QsSHORT VEGETATIVE PHASE 1*	QS116365.0	B, F Fr	36	6	Transcription factor, MADS-box
*QsSHORT VEGETATIVE PHASE 2*	QS055926.0	B, F Fr	9	23	Transcription factor, MADS-box

*Based on the analysis of the different transcription factor groups, a number of biological interesting genes were identified and their expression level was determined according to the number of ESTs in female (1F+2F) and male (1M+2M) Q. suber combined flower library Gene accessions according to CorkOak database (www.corkoakdb.org). Transcript distribution of the most differentially expressed genes in the Q. suber organs was obtained by blasting the genes sequences against the organ libraries made available by Pereira-Leal et al. (2014). R, root; L, leaf; B, bud; F, flower; Fr, Fruit*.

#### Zinc-finger TF family

The zinc-finger family of genes is an example of diversification in the Plant Kingdom and consists of a large number of proteins that are further classified into distinct subfamilies (Takatsuji, 1999). Among these, the C2H2-type and B-box zinc finger proteins constitute one of the largest families of transcriptional regulators in plants (Ciftci-Yilmaz and Mittler, 2008). Proteins containing zinc finger domains which play important roles in eukaryotic cells, regulating different signal transduction pathways and controlling processes such as development (Colasanti et al., 1998), homeostasis (Devaiah et al., 2007) and abiotic stress responses (Rizhsky et al., 2004; Sakamoto et al., 2004; Davletova et al., 2005). Some floral regulators contain a zinc-finger domain such as *CONSTANS* (*CO*), which has been linked to floral induction in several species by integrating the circadian clock and light signals (Putterill et al., 1995; Böhlenius et al., 2006). A clear *CO* homolog was not identified, as expected for the type of biological sample (flowers) used in the RNAseq. However, four *CO-like* transcripts differentially expressed in female flowers as compared to male flowers were identified. Of these, the *QsCONSTANS-LIKE 9* (*QsCOL9*) homolog was five times more expressed in female flowers. In *A. thaliana, COL9* delays flowering by reducing expression of *CO* and *FLOWERING LOCUS T* in leaves (Cheng and Wang, 2005). Its high level of expression in female tissues, particularly in early stages of the reproductive program, could suggest a novel function yet undisclosed. A homolog for the *A. thaliana* zinc-finger protein *TRANSPARENT TESTA 1* (*TT1*) was also seven times more expressed in female than in male *Q. suber* flowers. In melon, *CmWIP1* (a homolog of *TT1*) has a masculinizing effect by indirect repression of the ethylene driven *CmACS-7* gene (Boualem et al., 2008). *CmWIP1* needs to be epigenetically silenced to generate a fully functional female flower (Martin et al., 2009). The expression of *QsTT1* in the female flowers might point out the differences between developmental programs that give rise to sexual dimorphism in monoecious and dioecious/hermaphrodite species.

#### Basic helix-loop-helix (bHLH) TF family

The bHLH family encloses one of the largest groups of plants TF (Heim et al., 2003). These TF are involved in, among others, wound and stress responses (De Pater et al., 1997; Smolen et al., 2002; Chinnusamy et al., 2003; Kiribuchi et al., 2004), hormonal regulation (Abe et al., 1997; Friedrichsen et al., 2002) stigma and anther development, and fruit development and differentiation (Rajani and Sundaresan, 2001; Liljegren et al., 2004; Szécsi et al., 2006; Gremski et al., 2007). From the differentially expressed bHLH genes in *Q. suber* floral libraries, three were up-regulated in male flowers. One was the homolog of *ABORTED MICROSPORES*, a gene essential to the development of pollen (Sorensen et al., 2003; Xu et al., 2010). The other two *QsbHLH* are homologs to genes associated with iron deficiency (Wang et al., 2013). In female flowers, nine *Q. suber* transcripts were significantly more expressed, with one transcript being exclusive to the female samples (*QsBR ENHANCED EXPRESSION 1*). In *A. thaliana, BEE1* is involved in the brassinosteroids signaling and associated with the development of the reproductive tract (Crawford and Yanofsky, 2011). Homologs for *GLABRA3, MYC2, INDUCER OF CBF EXPRESSION1*, and *IAA LEUCINE RESISTANT3* were also up-regulated in female flowers. These genes are involved in hormonal regulation, cold acclimation, cell fate and double fertilization (Bernhardt et al., 2003; Chinnusamy et al., 2003; Yadav et al., 2005; Rampey et al., 2006; Dombrecht et al., 2007). However, there were three bHLH transcripts differentially expressed whose function is yet to be characterized in other species, making them good candidates at least to be involved in carpel development.

#### Basic leucine zipper (bZIP) TF family

The bZIP TFs regulate diverse biological processes in plants including flower development (Jakoby et al., 2002). Eight *Q. suber* bZIP associated genes were differentially expressed in female samples. Among them is the homolog of *VIP1*, an *A. thaliana* bZIP TF that regulates pathogen responses and rehydration responses (Tzfira et al., 2001; Tsugama et al., 2012). Another bZIP TF up-regulated in female samples is the homolog of *PERIANTHIA* (*PAN*), a gene involved in flower development in *A. thaliana* by altering floral organ number and initiation pattern (Running and Meyerowitz, 1996; Wynn et al., 2014). *PAN* is also involved in the activation of the C-class MADS box protein *AGAMOUS* (*AG*), a gene essential for carpel development (Das et al., 2009; Maier et al., 2009).

#### CUC/NAM TF family

Data also showed several *CUP-SHAPED COTYLEDON/NO APICAL MERISTEM* (*CUC/NAM*) genes highly represented in female flowers and the majority of them is differentially expressed. The *CUC/NAM* family encloses genes that control boundary formation and lateral organ separation, which are critical for proper leaf and floral patterning (Aida et al., 1997; Vroemen et al., 2003). A *CUC/NAM* gene in *Medicago trunculata* is needed for proper regulation of floral organ identity (Cheng et al., 2012). Also, in *A. thaliana, Petunia* and rice, mutants for *CUC/NAM* genes lead to the fusion of the cotyledons and some floral organs, as well as severe defects of the primary apical meristem (Souer et al., 1996; Aida et al., 1997; Mao et al., 2007). Out of *QsCUC/NAM* genes that are differentially expressed, homologs for *CUC1* and *CUC2* genes were up-regulated in female flowers.

#### MADS-box TF family

The MADS family of TF include a group of genes that play prominent roles in plant development (Smaczniak et al., 2012). Particularly, MADS TFs were found to be crucial for proper flower development in several species across the angiosperm lineage (reviewed in Theissen and Melzer, 2007). According to the canonical ABC model, which explains how homeotic genes control flower identity, stamens are formed by the activity of the B-Class and a C-class gene, whereas the same C-class is responsible for carpel development (Coen and Meyerowitz, 1991). As expected, B-class genes were differentially expressed in the male flowers (*QsAPETALA3* and *QsPISTILLATA*), and a similar level of expression of *QsAGAMOUS* (C-class gene) in both male and female libraries. The E-function genes (*SEPALLATA1-4*) that act as cadastral genes for proper organ development and identity (Pelaz et al., 2000) were also identified in both libraries but there was no sex differential expression. Several other homologs for MADS genes (Qs*APETALA1, QsFRUITFUL, QsFLOWERING LOCUS*, or *QsSUPRESSOR OF CONSTANS1*) that influence flowering in model and non-model species were also identified both in female and male libraries. Two transcripts similar to the *SHORT VEGETATIVE PHASE* gene were identified. It was interesting to detect a *QsSVP* gene differentially expressed in the female libraries and another in the male libraries. Genes of the *SVP* lineage in peach (*dormancy-associated genes, DAM*) are involved in growth cessation, bud set and break (Li et al., 2009).

### Hormone related genes differentially expressed in female and male flowers

Flower development is strongly affected by hormonal regulation (reviewed in Chandler, 2011). Auxin is tightly linked to the initiation of floral organ primordia and the disruption of auxin biosynthesis, polar auxin transport or auxin signaling leads to failure of flower formation (reviewed in Aloni et al., 2006). In agreement, the floral meristem identity gene *LEAFY* was recently shown to act through the regulation of the auxin response pathway (Li et al., 2013). Aux/IAA proteins, Auxin Efflux Carriers, and AUXIN RESPONSIVE FACTORS (ARF) are core components of the auxin-signaling cascade (Guilfoyle and Hagen, 2007). Several genes associated with the auxin regulatory network are highly represented and several are differentially expressed in female flowers. Particularly, homologs for the *A. thaliana IAA27* and *IAA9* genes were differentially expressed. The latter is a gene involved in fruit development and leaf embryogenesis in tomato (Wang et al., 2005) while mutants for *IAA27* showed altered fruit and flower development. A homolog of the *ARF4* gene that in *A. thaliana*, together with *ARF3*, control perianth organ number and spacing, as well as organ borders (Sessions et al., 1997) was identified in female flowers. Mutants for these genes showed defects in the stamens and gynoecium, as well as in the perianth organs, indicating an involvement in regional identity determination (Sessions et al., 1997). An auxin efflux carrier *QsPIN1* was also found to be up-regulated 20 fold in female flowers. Loss of *PIN1* function severely affects organ initiation; *pin1* mutants are characterized by an inflorescence meristem that does not initiate any flowers, resulting in the formation of a naked inflorescence stem (Okada et al., 1991). The abundance of auxin related machinery in female tissues leads to the possibility that female tissue determination might be under strong control of this hormone. Another hormone strongly correlated with sex determination is ethylene (Byers et al., 1972; Rudich et al., 1972). In cucumber, ethylene signaling is important in the inhibition of stamen development (Yamasaki et al., 2001). Interestingly, all the differentially expressed genes containing an ETHYLENE RESPONSIVE FACTOR (ERF) domain were detected in the female flowers, in agreement with the aforementioned role of ethylene in the feminization of the flower meristem. Another gene linked to ethylene signaling pathway is *EIN3*, a nuclear TF that initiates downstream transcriptional cascades for ethylene responses (Potuschak et al., 2003; Yanagisawa et al., 2003). *QsEIN3* was unique to the female samples. Interestingly in *Arabidopsis*, activated ethylene signaling reduces bioactive GA levels, thus enhancing the accumulation of DELLAs (repressors of the gibberellins pathway) and this most likely happens downstream of the transcriptional regulator *EIN3* (Achard et al., 2007). This is very interesting because the gibberellin hormone is thought to be essential for the developmental of a fully functional stamen in several species like *A. thaliana, Oryza sativa* or *Cucurbita maxima* (Pimenta Lange and Lange, 2006). This goes in agreement with our RNAseq results, in which several gibberellin related genes are found exclusively in the male flowers equally expressed in early and late stages of the developmental program, indicating a role in floral primordia and anther differentiation. Also, a *GAMYB* firmly involved in anther development, *QsMYB33*, was only expressed in male samples (Millar and Gubler, 2005). Interestingly, all the GRAS associated transcripts (known to be important regulators in GA signaling) that includes the gibberellin repressors DELLA, were only present in the female database.

## Conclusion

Monoecious and dioecious species have been long considered unique tools to study the developmental programs involved in the formation of separate male and female flowers. However, for the majority of these species, insufficient or inexistent genomic and transcriptomic data availability has hampered functional studies. Advances in NGS technologies have made possible to perform a rapid and cost-effective compilation of large RNA sequence data sets in non-model organisms with no or little prior genomic data available. Here, a broad flowering transcriptome composed of four independent libraries was obtained for early and late developmental stages of male and female flowers of *Q. suber*, a monoecious tree. In the future, to further enhance our knowledge on sex-specific genetic networks, individual EST libraries could be obtained for each phenological stage to fine map male and female flower specific regulators. Comparative studies revealed a subset of transcripts that were differentially expressed in the different libraries, many of which have a known role in flower and/or plant development. Transcriptome analysis also revealed a group of genes expressed exclusively in each type of flower gender that may have a functional role in male and female flower organ development or in sex specification. Some of the genes that showed differential expression have not been previously characterized in other species and others have not, to our knowledge, been implicated in flower development. Thus, it would be very interesting to perform functional studies using the above mentioned genes to identify its roles in plant reproduction or flower development in *Q. suber* and other flowering species. The analysis of *Q. suber* flower transcriptome may therefore contribute to uncover sex-specific regulatory networks hidden by hermaphroditism and serve as a platform to future studies in model and non-model species independently of their sexual habit.

## Author contributions

Leonor Morais-Cecílio, Maria M. R. Costa—Conceived and designed the experiments. Margarida Rocheta, Rómulo Sobral, Maria I. Amorim, Teresa Ribeiro, Leonor Morais-Cecílio, Maria M. R. Costa—Preparation of plant material and RNA. Margarida Rocheta, Rómulo Sobral, Joana Magalhães, Maria I. Amorim, Leonor Morais-Cecílio, Maria M. R. Costa—Performed the experiments. Miguel Pinheiro, Conceição Egas—Transcriptome sequencing and Bioinformatics. Margarida Rocheta, Rómulo Sobral, Joana Magalhães, Maria I. Amorim, Miguel Pinheiro, Leonor Morais-Cecílio, Maria M. R. Costa—Data analysis. Margarida Rocheta, Rómulo Sobral, Joana Magalhães, Maria I. Amorim, Miguel Pinheiro, Leonor Morais-Cecílio, Maria M. R. Costa—Paper writing and discussion. All authors read and approved the final manuscript.

### Conflict of interest statement

The authors declare that the research was conducted in the absence of any commercial or financial relationships that could be construed as a potential conflict of interest.

## Supplementary material

Figure S1 **Functional classification of *Quercus suber* differentially expressed unigenes**.

Table S1 **List of PCR primers used to amplify candidate genes**.
