# Supplementary material for: Comparative transcriptomic analysis of male and female flowers of monoecious Quercus suber
Source: Front Plant Sci. 2014 Nov 6;5:599. doi: 10.3389/fpls.2014.00599 (PMC4222140; doi:10.3389/fpls.2014.00599)
Supplement: Figure S1 — Functional classification of Quercus suber differentially expressed unigenes. [file Image1.PDF]

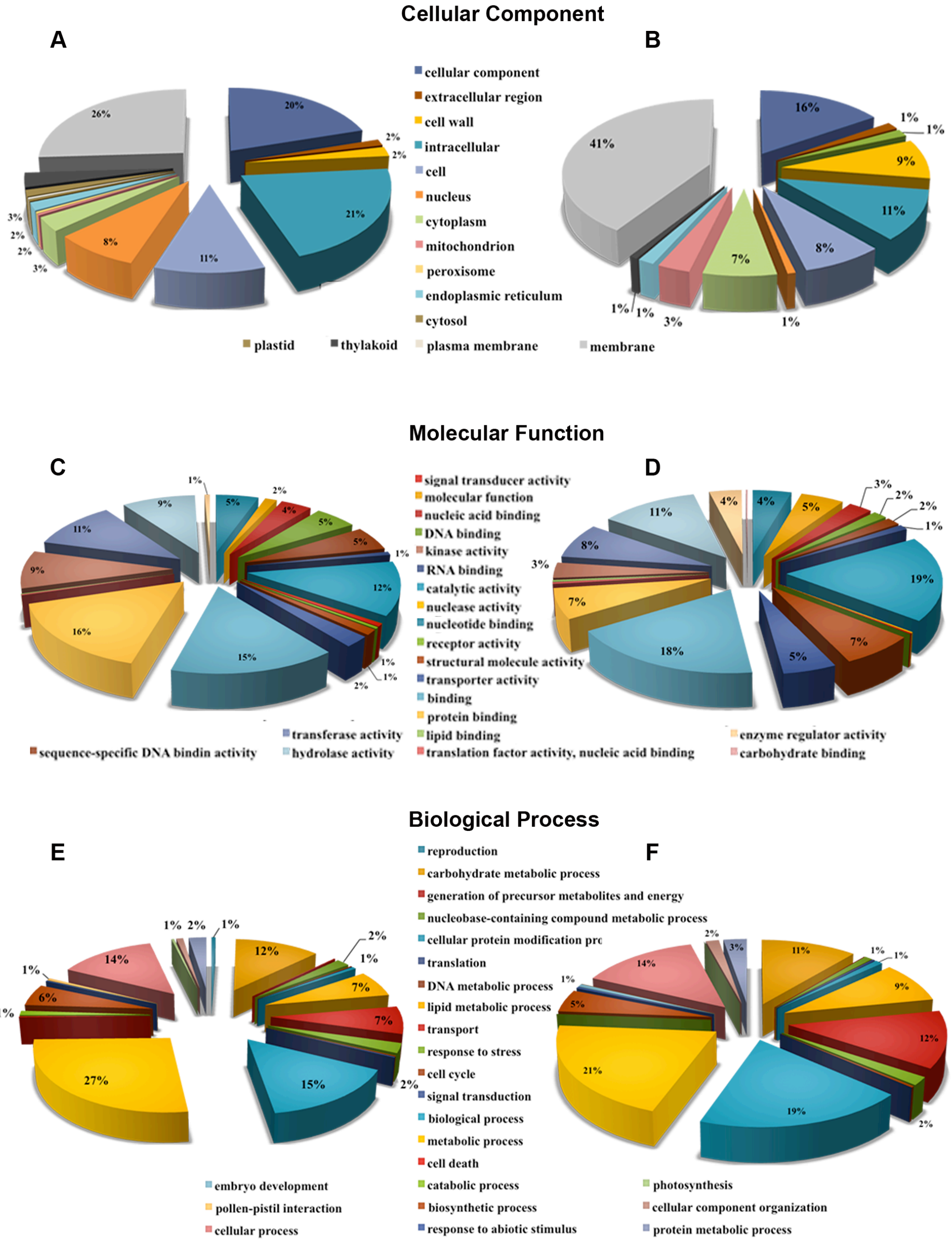

Figure S1. Functional classification of *Q. suber* differentially expressed unigenes. The differentially expressed genes in male and female libraries were identified by normalization at a 95 percentile using the MyRNA statistical analysis package. The female (A), (C) and (E) and male (B), (D) and (F) differentially expressed genes were classified within the category of cellular component, molecular function and biological process using Gene Ontology terms (GOs) for each translated sequence.
