# Supplementary material for: Comparative transcriptomic analysis of male and female flowers of monoecious Quercus suber
Source: Front Plant Sci. 2014 Nov 6;5:599. doi: 10.3389/fpls.2014.00599 (PMC4222140; doi:10.3389/fpls.2014.00599)
Supplement: Table S1 — List of PCR primers used to amplify candidate genes. [file Table1.DOCX]

**Supplementary Table 1 -** List of the PCR primers used to amplify the candidate genes that were selected to validate the transcriptional levels determined by RNA sequencing results.

| **Candidate gene in model species** | **Primer F** | **Primer R** | **Fragment Length** |
| --- | --- | --- | --- |
| *At4g27290* | CGATTGAGCTGCGAGGAGA | CAGCCAGCACCTTCTCTGA | 182 bp |
| *CYTOCHROME P450 78A9* | TTTACTTGCCAGCTGTGGTG | GACCCCATGACAGAAAACTC | 244 bp |
| *POLYGALACTURONASE1* | AGCGGGAAAGAGATGCAACA | TGACAAACCTCGATCCGACC | 239 bp |
| *STIGMA SPECIFIC1* | GGGTTTGCCATGCTAAGAGC | AGCTGCACATCCCATACACA | 225 bp |
| *ABORTED MICROSPORES* | CACAACAGATGGAGGTGCAA | AAGCAAGGAGTCCCTCACA | 242 bp |
| *LESS ADHERENT POLLEN3* | AACCACCAACTGCAGGCTAA | CTCATTCGGATTGGTAACCG | 219 bp |
| *LESS ADHESIVE POLLEN5* | AGCTTCACACAGCAATCCAG | CTCAAGTCGCTTTTCCATCC | 248 bp |
| *LESS ADHESIVE POLLEN6* | ATTGGAGCGCCTGTGCAAAA | TCCCGGTAAGCGTATTTCAC | 253 bp |
| *PROTEIN PHOSPHATASE 2A SUBUNIT A3* | GGGTTCCCAACATCAAGTTC | TGACCTGATCACTTGACTGC | 174 bp |
